# Effects of Sugar Impregnation Methods on Physicochemical Properties and Flavor Profiles of Prune Preserves Using GC-IMS and Electronic Tongue

**DOI:** 10.3390/foods14162852

**Published:** 2025-08-18

**Authors:** Qingping Du, Rui Yang, Wei Wang, Wei Li, Tongle Sun, Shihao Huang, Xinyao Han, Mingxun Ai

**Affiliations:** College of Food Science and Pharmacy, Xinjiang Agricultural University, Urumqi 830052, China; dqp1885660@163.com (Q.D.);

**Keywords:** prune preserves, sugar impregnation methods, volatile organic compounds, quality

## Abstract

Thermal impregnation (TI) is a traditional method of sugar infusion, but it has disadvantages such as long processing time and uneven sugar distribution. Therefore, developing sugar impregnation methods to enhance product flavor, nutritional value, and processing efficiency is critical for addressing potential quality loss and efficiency bottlenecks in traditional preserve processing technologies. This study took the TI process widely adopted in Xinjiang over the long term as a reference and systematically compared the effects of vacuum impregnation (VI) and ultrasonic-assisted impregnation (UI) on the flavor characteristics and physicochemical properties of plum preserves. Volatile organic compounds (VOCs) were identified using gas chromatography–ion mobility spectrometry (GC-IMS) coupled with multivariate analysis, while taste attributes were quantified via electronic tongue (E-tongue). Physicochemical parameters, including titratable acidity (TA), browning index (BI), color parameters (L*, a*, b*), total polyphenol content (TPC), total flavonoid content (TFC), and texture profile analysis (TPA), were also evaluated. GC-IMS identified 60 VOCs, predominantly comprising aldehydes (20), alcohols (10), ketones (6), acids (4), esters (3), furans (3), ketols (2), and unidentified compounds (12). The VI-treated samples exhibited distinct aromatic profiles, retaining a higher proportion of key volatile compounds. E-tongue results showed that VI significantly enhanced sourness, umami, and aftertaste complexity compared with UI and TI (*p* < 0.05). Physicochemical analyses showed that VI maximally preserved bioactive compounds, with a TPC of 1.23 ± 0.07 mg GAE/g and TFC of 17.55 ± 0.81 mg RE/g. Additionally, VI minimized enzymatic browning (BI: 0.37 ± 0.03), maintained color brightness (L*: 31.85 ± 1.56), maintained favorable textural properties (hardness: 187.63 ± 4.04 N), and retained the highest TA content (0.77 ± 0.05%). In contrast, UI and TI led to significant quality degradation, characterized by pronounced browning and texture deterioration: the BI values were 0.61 ± 0.02 (UI) and 0.83 ± 0.03 (TI), and hardness values were 176.53 ± 5.81 N (UI) and 156.25 ± 4.55 N (TI). These findings provide critical references for sugar impregnation techniques and a scientific basis for flavor regulation in prune preserve production.

## 1. Introduction

Plums (*Prunus domestica* L.), which belong to the Rosaceae family and are native to Central and Western Asia [1], are renowned for their rich nutritional profile and exhibit antioxidant and immunomodulatory properties [2]. However, the high water content of fresh plums leads to rapid post-harvest deterioration, severely limiting their commercial value and market circulation. Therefore, to extend their shelf life and effectively preserve their precious nutritional components, processing them into preserves has become an important solution. Preserves are semi-dried foods made from fresh fruits through processes such as sugar impregnation and drying. Prune preserves are celebrated for their high fiber content, laxative effects, and antioxidant properties, positioning them as portable snacks combining gut health benefits with natural sweetness. Nevertheless, traditional drying processes incur substantial energy consumption [3]. To address this challenge and enhance drying efficiency and product quality, various predrying technologies have been adopted, with osmotic dehydration gaining prominence for its energy-saving potential and ability to maintain fruit characteristics [4].

Sugar impregnation modifies the physicochemical properties of fruits and vegetables through solute osmosis and is a core process in food processing [5]. The efficacy of the sugar impregnation process depends on the specific processing method. As the predominant preserve production method for decades, traditional thermal impregnation (TI) persists in widespread use due to its operational simplicity and minimal equipment requirements [6]. TI employs heated sugar solutions (50–70 °C) to drive diffusion-based solute transfer [7]. This simple soaking process relies exclusively on thermal energy without external physical forces, resulting in inherent limitations: prolonged processing, thermolabile compound degradation, and non-uniform sugar distribution [8]. These limitations have prompted the exploration of alternative technologies such as vacuum impregnation (VI) and ultrasonic-assisted impregnation (UI). VI is a process involving two distinct phases: First, negative pressure evacuates gases and liquids from the porous structure of plant tissues. Subsequently, upon restoration of atmospheric pressure, the pressure differential forces the external solution into the evacuated pores, ensuring uniform solute distribution within the tissue [9]. UI utilizes high-frequency mechanical waves (>20 kHz) to enhance pretreatment [10]. Through cavitation effects, UI improves the permeability of fruits and vegetables immersed in hypertonic solutions, significantly boosting sugar impregnation efficiency and accelerating subsequent drying [11]. However, the denser microstructure and lower intrinsic porosity of prune tissues necessitate micro-perforation pretreatment to enhance permeability and achieve adequate solute infusion during impregnation. Compared with TI, UI not only significantly shortens the drying time of Sanhua plums (*Prunus salicina* L.) but also enhances drying efficiency, polyphenol retention, antioxidant capacity, and sensory attributes (e.g., texture softening, color stability) [3,12]. VI has been shown to significantly influence the flavor and color of prune preserves [13].

Flavor, a key sensory attribute determining preserve quality and consumer acceptance, arises from the synergistic effect of volatile aroma compounds and non-volatile taste substances [14]. During thermal processing, flavor evolution is driven by two primary mechanisms: firstly, biochemical pathways such as the Maillard reaction and lipid oxidation reshape the profile of flavor compounds at the molecular level, generating characteristic aromatics (e.g., furans, aldehydes) while promoting the degradation of heat-sensitive components (e.g., terpenes); concurrently, structural degradation mechanisms, including cell wall breakdown and membrane permeability changes, enhance enzyme–substrate contact and proton transfer rates, accelerating the release or loss of flavor precursors [15,16]. Deciphering these complex flavor dynamics requires a multi-dimensional technical approach. GC-IMS, with its high-throughput, non-destructive, and highly sensitive features, enables real-time capture of trace VOCs (e.g., the dynamic decay of hexanal during thermal processing) without complex sample pretreatment, providing a theoretical basis for identifying key flavor markers [17]. Concurrently, the E-tongue system quantifies non-volatile taste substances (e.g., organic acids, amino acids, nucleotides) by simulating biological taste mechanisms, serving as an effective means to supplement the subjectivity of traditional sensory evaluation. Importantly, taste parameters output by the E-tongue (e.g., changes in sourness or umami) can be cross-validated with physicochemical indices (e.g., TA), enhancing the objectivity and reliability of data interpretation [18]. On this basis, textural properties, as another critical dimension in sensory perception, not only determine the masticatory experience of food but also influence the release and diffusion of flavor compounds in saliva [19]. TPA measures physical responses (e.g., hardness, elasticity, adhesiveness) by simulating the compression–relaxation cycle of chewing, thereby deciphering the “mechanistic contribution” of tissue structure to flavor release. For instance, a study by Yu et al. on chestnut matrices has demonstrated that TPA parameters can effectively reflect oral sensory attributes, establishing a complementary relationship with E-tongue data [20]. Therefore, cross-modal fusion analysis of physical indices (e.g., TPA, TA) with GC-IMS and E-tongue data helps reveal the causal chain from microstructural changes to macroscopic sensory expression, providing a scientific basis for precise flavor regulation and multi-dimensional quality optimization in prune preserve processing.

Current research predominantly focuses on isolated analyses of single quality indices (e.g., texture or color), lacking systematic evaluations that integrate modern flavor omics (GC-IMS) with artificial taste simulation (E-tongue). Importantly, the mechanistic interactions among VI, UI and TI technologies in regulating the synergistic evolution of flavor chemical profiles and physicochemical matrix in prune preserves remain a critical unaddressed gap in current scientific understanding. Therefore, this study investigates the effects of three impregnation methods on the quality of French prune preserves, including color parameters (L*, a*, b*), BI, TA, TPC, TFC, and TPA. And flavor compounds and fingerprint profiles were characterized using E-tongue and GC-IMS technologies. Thereby, the optimal sugar impregnation method was screened out to enhance the taste, flavor, texture, and overall acceptability of French prune preserves, thus improving consumer satisfaction. This study not only provides a reference for the optimization of prune preserve processing technology but also lays an empirical foundation for subsequent research on flavor characteristic regulation of prune preserves.

## 2. Materials and Methods

### 2.1. Materials

In September 2024, French plums (*Prunus domestica* L.) with a diameter of 3.5–4.0 cm, at 80% maturity, purplish-red color, and free of mechanical damage or mildew were procured from Kashgar, Xinjiang, China. Fresh fruits were stored at 4 °C for 7 days to stabilize their ripening degree. The initial total soluble solids (TSSs) of the raw fruits were 18.7 ± 0.5 °Brix (*n* = 10). All chemical reagents and solvents used in this study were of analytical grade, purchased from Solarbio Science & Technology Co., Ltd., Beijing, China.

### 2.2. Sample Preparation

Sound French plums were selected, washed, pitted, and subjected to color protection and hardening treatment in 0.1% CaCl_2_ solution for 20 min, followed by draining for subsequent use. The pretreated plum fruits were evenly divided into three groups (each serving as a treatment unit, containing at least 10 fruits). After uniform micro-perforation using needles sterilized with 75% ethanol, the fruits were immersed in a hypertonic 35% (*w*/*w*) mixed sugar solution (sucrose–erythritol = 1:2, *w*/*w*) for sugar impregnation. This high-concentration sugar solution—markedly higher than the initial TSSs of the raw material (18.7 ± 0.5 °Brix)—was selected based on preliminary experiments to establish a strong osmotic driving force, thereby facilitating dehydration and solute uptake. The sucrose–erythritol ratio (1:2) was chosen to leverage the low-calorie property of erythritol as well as its advantage of providing higher molar concentration and osmotic pressure at a given concentration due to its lower molecular weight compared with sucrose. This was followed by sugar impregnation using three methods: VI, UI, and TI. Parameters for VI, UI, and TI were determined via one-way ANOVA based on preliminary single-factor experiments. These experiments evaluated the effects of sugar impregnation time, temperature, pressure, and ultrasonic power on key quality attributes including moisture content, hardness, and color retention. VI group was impregnated for 30 min at 0.07 MPa using ZG10 vacuum sugar impregnation equipment (Zhucheng Dehua Machinery Factory, Weifang, China), followed by 8 h of atmospheric equilibration at room temperature [21,22]. UI group was impregnated for 70 min at 300 W using KQ-300TDE ultrasonic instrument (Kunshan Ultrasonic Instruments Co., Ltd., Kunshan, China), followed by 8 h of atmospheric equilibration at room temperature [23,24]. TI group was impregnated for 70 min at 60 °C using a DZKW-D-2 thermostatic water bath (Beijing Yongguangming Medical Instrument Factory, Beijing, China), followed by 10 h of atmospheric equilibration at room temperature [7]. Following impregnation, samples were left to equilibrate at atmospheric pressure at room temperature for 8 h (VI, UI) or 10 h (TI). This equilibration step is essential to allow uniform solute distribution and tissue rehydration before drying, helping reduce concentration gradients and preparing the tissue for consistent drying. All sugar-impregnated samples (VI, UI, TI) were dried in the same batch in a DHG-9070A hot-air drying oven (Qixing Scientific Instruments Co., Ltd., Shanghai, China) at 50 ± 1 °C with an air velocity of 1.5 m/s until reaching a target moisture content of 15–20% (wet basis) to eliminate the influence of drying batch differences. Moisture content was determined using a DHS-16 moisture analyzer (Shanghai Jinghai Instrument Co., Ltd., Shanghai, China) following the method described in the literature [25]. A completely randomized design was employed in this experiment, where all sugar impregnation treatments (VI, UI, TI) were independently replicated three times (*n* = 3). Each replication utilized an independent treatment unit (i.e., a batch of raw fruits), and all analyses were conducted based on data from three independent replicate experiments. All physicochemical, structural, and sensory analyses were performed on dried samples.

### 2.3. Solute Uptake Determination

To quantitatively evaluate the impregnation efficiency, solute uptake was determined by measuring the TSSs (expressed as °Brix) and a_w_ of the drying samples. TSS measurement: the method of Dayang Liu et al. [26] was referenced. The determination was performed using a PAL-1 digital refractometer (Atago Co., Ltd., Tokyo, Japan). The specific procedures were as follows: Obtain the prune preserves tissue. Weigh precisely 10 g of the sample and add 50 mL of deionized water. Vortex and oscillate for 30 min to ensure the full dissolution of TSSs. After filtering through Whatman No. 4 filter paper, collect the supernatant for measuring the sugar content. Each group was replicated three times. A_w_ measurement: the protocol of Xie JW et al. [27] was followed. A_w_ was determined using an AquaLab 4TE water activity meter (Decagon Devices Inc., Pullman, WA, USA) at 25 °C. The instrument was calibrated with saturated NaCl solution (a_w_ = 0.753) prior to measurements. Each sample was analyzed in triplicate.

### 2.4. Determination of TA

TA was determined using the first-method acid–base indicator titration according to Paul’s protocol [28]. Specifically, 10 g of homogenized prune preserve sample (particle size < 2 mm) was accurately weighed, dissolved in deionized water, and then adjusted to a final volume of 100 mL. The mixture was shaken at room temperature for 30 min, then filtered through Whatman No. 4 filter paper. A 20 mL aliquot of the filtrate was transferred to a flask, added with 2 drops of 1% phenolphthalein indicator, and titrated with 0.1 mol/L NaOH standard solution to a faint pink color that persisted for 30 s. The volume of NaOH consumed was recorded, with three replicates performed. A blank titration using distilled water instead of the filtrate was conducted for correction. TA was calculated using the following formula:

(1) $$TA\left(\%\right)=\frac{V\times c\times (V_{1}-V_{0})\times 0.064}{m\times V_{S}}\times 100\%$$

where *V* is the total volume of the sample extract (mL); *V_s_* is the volume of filtrate taken for titration (mL); *c* is the concentration of the NaOH titrant (mol/L); *V*_1_ is the volume of NaOH solution consumed for titrating the filtrate (mL); *V*_0_ is the volume of NaOH solution consumed for titrating distilled water (mL); *m* is the sample mass (g); 0.064 is the conversion coefficient calculated using citric acid.

### 2.5. Spectrophotometric Analysis 

#### 2.5.1. Determination of BI and Color Measurement

The method described by Lee was adapted with minor modifications [29]. Two grams of dried powder sample was accurately weighed and thoroughly mixed with 15 mL of 95% ethanol. The mixture was centrifuged at 8000 r/min for 10 min using a 3H16RI centrifuge (Hunan Hexi Instrument Equipment Co., Ltd., Changsha, China). The supernatant was collected, and its absorbance was measured at a wavelength of 420 nm using a P4PC UV–visible spectrophotometer (Shanghai Mapada Instruments Co., Ltd., Shanghai, China). All experiments were performed in three independent replicates. BI was calculated using the following formula:

(2) $$BI=\frac{A_{420}\times D}{W}$$

where *D* is the dilution factor; *W* is the sample mass (g); *A*_420_ is the absorbance at 420 nm.

Color parameters (L*, a*, b*) were measured using a CR-10Plus spectrophotometer (3NH Technology Co., Ltd., Zengcheng, China) [30]. The instrument was calibrated with standard white and black tiles before each measurement. Surface values for L*, (lightness), a* (redness), and b* (yellowness) were recorded, with three replicate measurements per sample.

#### 2.5.2. Determination of TPC and TFC

TPC was determined using the Folin–Ciocalteu method [31]. One milliliter of sample extract, prepared by homogenizing the sample with 80% methanol at a ratio of 1:10 (*w*/*v*) for 2 h followed by centrifugation at 8000 r/min for 10 min using a 3H16RI centrifuge (Hunan Hexi Instrument Equipment Co., Ltd., Changsha, China), was taken. Five milliliters of 10% Folin–Ciocalteu reagent was added to the extract, and the mixture was vortexed for 5 min. Subsequently, 4 mL of 7.5% Na_2_CO_3_ solution was added, and the mixture was incubated in a 75 °C water bath for 10 min. After cooling, the absorbance was measured at 760 nm using a P4PC UV–visible spectrophotometer (Shanghai Mapada Instruments Co., Ltd., Shanghai, China). Results were expressed as milligrams of gallic acid equivalents per 100 g of dry matter (mg GAE/100 g DM).

TFC was assayed according to Ghasemzadeh et al. [32] using the same extract. Absorbance was measured at 325 nm, and quantification was performed using a rutin standard curve (0–100 μg/mL), with results expressed as rutin equivalents (mg RE/100 g DM).

### 2.6. Measurement of Texture

TPA was performed according to the method of Zahari et al. [33] using an XTPlus texture analyzer (Beijing Weixun Chaoji Technology Co., Ltd., Beijing, China) equipped with a P/36R cylindrical probe (diameter 36 mm). The measurement parameters were set as follows: pre-test speed 2 mm/s, test speed 0.5 mm/s, trigger force 5 g, return speed 1 mm/s, compression deformation rate 20%, and recovery waiting time 5 s. Hardness is defined as the peak force of the first compression (N), reflecting the initial compressive strength of the sample. Springiness refers to the ratio of the height of the second compression to that of the first compression (dimensionless), which characterizes the deformation recovery ability. Cohesiveness is the ratio of the positive work of the second compression to that of the first compression (dimensionless), reflecting the internal binding force. Gumminess, calculated as hardness multiplied by cohesiveness (N), represents the chewing resistance of semi-solid samples. Chewiness, calculated as gumminess multiplied by springiness (mJ), characterizes the energy required to chew solid samples until they are ready for swallowing. Each sample was measured in triplicate.

### 2.7. GC-IMS Analysis of Volatile Compounds

The method described by Zheng [34] was adapted with minor modifications. VOCs in prune preserves were analyzed using a FlavourSpec^®^ (Gesellschaft für Analytische Sensorsysteme GmbH, G.A.S., Dortmund, Germany), equipped with an MXT-5 capillary column (15 m × 0.53 mm × 1.0 μm, Restek Corporation, Bellefonte, PA, USA) and a CTC-PAL 3 automatic headspace sampling device (CTC Analytics AG, Zwingen, Switzerland). Sample preparation: 2.00 ± 0.01 g of ground dried sample was placed in a 20 mL headspace vial and equilibrated at 60 °C with shaking (500 r/min) for 15 min, and 500 μL of headspace gas was extracted for injection (splitless, inlet temperature 85 °C). The chromatographic conditions were as follows: injector temperature, 80 °C; isothermal operation of the MXT^®^-5 column at 60 °C; high-purity nitrogen (≥99.999%) as the carrier gas with a programmed flow rate consisting of an initial flow rate of 2.0 mL/min held for 2 min, increased to 10.0 mL/min over 8 min, further increased to 100.0 mL/min over 10 min, and finally increased to 150.0 mL/min over another 10 min (total run time of 30 min); the column was connected to the IMS drift tube via a transfer line maintained at 100 °C. The IMS drift tube temperature was 45 °C, carrier gas (N_2_) flow rate was 150 mL/min, and drift gas (N_2_) flow rate was 50 mL/min. VOCs were identified by comparing retention indices (RIs) and drift times against the NIST 2017 database and the built-in G.A.S. library for qualitative analysis.

### 2.8. Determination of E-Tongue

The measurement was performed using an Insent SA402B E-tongue (Tokyo, Japan) with sensors for detecting acids, sugars, umami, etc., following the method of Bai [35] with minor modifications. For sample preparation, 5 g of dried sample was homogenized with 50 mL of ultrapure water, centrifuged at 4000 r/min for 10 min, and filtered through a 0.45 μm membrane prior to measurement. Sensors were activated with 3 mm KCl solution for 30 min before analysis. Each measurement used 20 mL of sample, with a 60 s equilibration time, 120 s response time, and 90 s cleaning time, repeated three times. Data were processed via pattern recognition software.

### 2.9. Statistical Analysis

All treatments and analyses were performed using independent biological replicates. Each experiment was repeated at least three times independently (*n* = 3). Statistical analyses were conducted using IBM SPSS Statistics 23, including Waller–Duncan multiple range tests (*p* < 0.05), and results are presented as mean ± standard deviation. Origin 2021 was used for graphing, while SIMCA 14.1 facilitated principal component analysis (PCA), orthogonal partial least squares discriminant analysis (OPLS-DA), and variable importance in projection (VIP) analysis. Model reliability was validated via 200-cycle permutation tests.

## 3. Results and Discussion

### 3.1. Analysis of Solute Uptake

As shown in Table 1, VI treatment achieved a significantly higher TSS of 30.10 ± 0.26 °Brix (*p* < 0.05). The VI group exhibited a significantly higher TTS than the UI and TI groups (*p* < 0.05), indicating that VI more effectively promoted the penetration of solutes (e.g., sugars) into the plum tissues. Despite prunes’ denser structure and lower porosity than apples or pears [36], VI achieved superior sugar penetration via dual mechanisms: First, micro-perforation pretreatment created auxiliary solute channels (Section 2.2). Second, the negative pressure phase (0.07 MPa) not only evacuated air from existing pores but also induced microstructural changes in cell walls and intercellular spaces, temporarily increasing effective porosity during processing [37]. This aligns with findings that pretreatment overcomes low-porosity limitations [36], and VI induces tissue restructuring to facilitate impregnation [38]. In contrast, the TI group relied on temperature-driven diffusion, and prolonged high temperatures might have caused partial sugar degradation, resulting in the lowest TSS. The a_w_ showed an inverse trend to TSS: the VI group had the lowest a_w_ (0.86 ± 0.01), significantly lower than the UI (0.91 ± 0.01) and TI (0.93 ± 0.01) groups (*p* < 0.05). This is because the higher sugar content in the VI group reduced the proportion of free water through solute–water interactions, which can effectively inhibit microbial growth and extend product shelf life, consistent with the processing objectives of dried fruit products [39]. The TSS of commercial prune preserves is usually 28–35 °Brix [40]. In this study, the 30.10 ± 0.26°Brix of the VI treatment was closer to this range, while the lower sugar content of the UI and TI treatments might result in insufficient sweetness of the product, which could affect market acceptance [41].

### 3.2. TA Analysis

**Figure 1:**
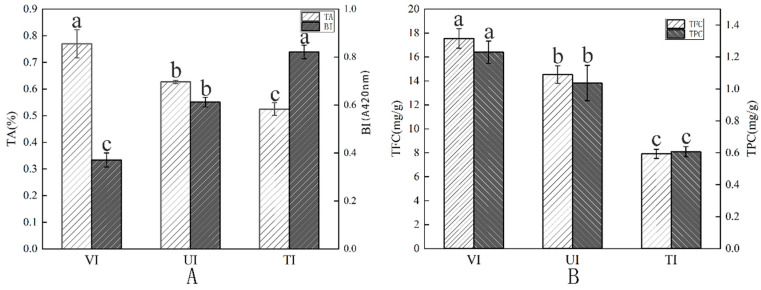
Effects of different sugar impregnation methods on TA and BI (**A**) as well as TPC and TFC (**B**) in prune preserves. For each parameter tested, different letters denote significant differences at *p* < 0.05.

TA, a key determinant of fruit flavor composed of various organic acids, is profoundly influenced by processing techniques [42]. As shown in Figure 1A, significant differences (*p* < 0.05) in TA retention were observed among prune preserves by different sugar impregnation methods: VI (0.77 ± 0.05%) > UI (0.63 ± 0.01%) > TI (0.53 ± 0.03%). This discrepancy primarily stems from the distinct characteristics of each impregnation process. The VI group featured a relatively short, intense active impregnation period (30 min), which likely limited acid leaching and thus resulted in the highest TA retention, whereas longer soaking in UI and TI increased diffusion-driven acid loss. [43]. Temperature emerges as a critical factor affecting TA retention [44]. In the UI treatment, ultrasonic cavitation induced an instantaneous high-temperature and high-pressure field, accelerating the thermal degradation of heat-sensitive organic acids (e.g., citric acid, malic acid), thereby reducing the TA content [45]. By contrast, the TI group showed the lowest TA content due to prolonged high-temperature treatment causing acid degradation. The higher TSS in VI-treated samples enhances sweetness perception and may moderate the taste balance, softening perceived acidity despite similar TA levels. This sweetness–acidity interaction aligns with E-tongue results showing VI samples had the highest sourness intensity but balanced acceptability, likely due to the elevated sugar content compensating for the higher TA [46].

### 3.3. Spectrophotometric Analysis

#### 3.3.1. BI and Color Measurement Analysis

Color is a critical determinant of preserved fruit quality, significantly influencing market acceptability [13]. The browning process in preserved fruits initiates with enzymatic reactions and shifts to non-enzymatic browning (Maillard reaction and caramelization) under thermal processing [47]. Our results show that VI treatment significantly inhibited the prune preserves browning. As depicted in Figure 1A, the BI of the VI group was 0.37 ± 0.03, markedly lower than that of the UI (0.61 ± 0.02) and TI (0.83 ± 0.03) groups, representing approximately 39.34% and 55.42% reductions, respectively. As shown in Table 1, L* values were 31.85 ± 1.56 (VI), 28.05 ± 0.83 (UI), and 25.17 ± 0.85 (TI), indicating that VI best maintained color brightness. Additionally, the VI group exhibited the highest redness (a*) value (17.03 ± 0.58 > 12.88 ± 0.72 > 11.13 ± 0.95), reflecting more pronounced red hues. This phenomenon is attributed to the hypoxic environment in VI, which suppresses polyphenol oxidase (PPO) activity and reduces substrate exposure for Maillard reactions [48]. Conversely, ultrasonic cavitation in UI disrupts the spatial structure of PPO (e.g., breaking disulfide bonds in the active center), causing irreversible enzyme inactivation and thereby decreasing browning [49]. The high temperature in TI accelerates pigment degradation and melanoidin formation, leading to color darkening [50]. Overall, VI best preserves the natural color of prune preserves, consistent with visual observations.

#### 3.3.2. TPC and TFC Analysis

As key bioactive compounds in prune preserves, TPC and TFC determine the antioxidant capacity, sensory quality, and nutritional value [51]. As illustrated in Figure 1B, the VI treatment remarkably outperformed other methods in preserving bioactive components. The VI group exhibited a TPC of 1.23 ± 0.07 mg GAE/g and a TFC of 17.55 ± 0.81 mg RE/g, whereas the UI group showed significantly lower TPC (1.04 ± 0.11 mg GAE/g) and TFC (14.53 ± 0.74 mg RE/g). The TI group had the lowest TPC (0.61 ± 0.32 mg GAE/g) and TFC (7.91 ± 0.39 mg RE/g). This superiority stems from the dual protective effects of the VI process: First, room temperature (25 ± 2 °C) and 0.07 MPa negative pressure significantly inhibited polyphenol oxidase activity and delayed phenolic auto-oxidation [52]. Second, the vacuum pressure difference (confirmed by higher TSS in VI) likely accelerated sugar solution penetration; elevated sugar levels likely act as osmotic protectants, reducing phenolic compound degradation during drying and contributing to higher retention in VI samples, reducing cell structure damage and minimizing the loss of TPC and TFC [53,54]. In contrast, the transient extreme conditions and strong oxidative radicals generated by ultrasonic cavitation in the UI group synergistically induced thermal degradation and oxidative bond cleavage of TPC and TFC, leading to lower retention rates than VI [55]. Prolonged thermal exposure in TI caused phenolic degradation, resulting in significantly reduced TPC and TFC [56]. Notably, phenolic compounds not only serve as substrates for Maillard reactions but also directly regulate the color, taste, and flavor characteristics of prune preserves [57]. Thus, VI-processed prune preserves combine higher antioxidant activity with lower browning degree, further demonstrating the superior performance of VI in maintaining the nutritional and sensory properties of prune preserves.

### 3.4. Texture Analysis

TPA quantitatively characterizes the eating texture and structural integrity of prune preserves, encompassing key parameters such as hardness, adhesiveness, cohesiveness, gumminess, and chewiness [58]. Among these, chewiness is the primary determinant of consumer acceptability [59]. As shown in Table 1, the VI treatment significantly enhanced the textural properties of prune preserves: hardness (187.63 ± 4.04 N) and chewiness (113.99 ± 6.61 mJ) were notably higher than those in the UI group (hardness 176.53 ± 5.81 N, chewiness 93.52 ± 5.70 mJ) and TI group (hardness 156.25 ± 4.55 N, chewiness 69.56 ± 4.65 mJ). This advantage stems from the unique mechanism of VI: the negative-pressure stage (0.07 MPa) mainly serves to expel air within the tissue, creating a vacuum in the intercellular spaces; during the subsequent atmospheric pressure recovery stage, the external sugar solution rapidly fills the pores and penetrates into the intercellular spaces driven by the pressure difference; higher TSS may promote sugar–pectin interactions, promoting the formation of a three-dimensional sugar–pectin–cellulose network that maintains cell structural integrity [60]. In contrast, prune preserves treated with TI and UI exhibited softened flesh tissues due to thermal degradation (high temperature) and cavitation damage (mechanical force), respectively, consistent with their significantly reduced hardness, chewiness, cohesiveness, and gumminess [61].

### 3.5. GC-IMS Analysis

#### 3.5.1. GC-IMS Profiling and Key VOCs Identification

**Figure 2:**
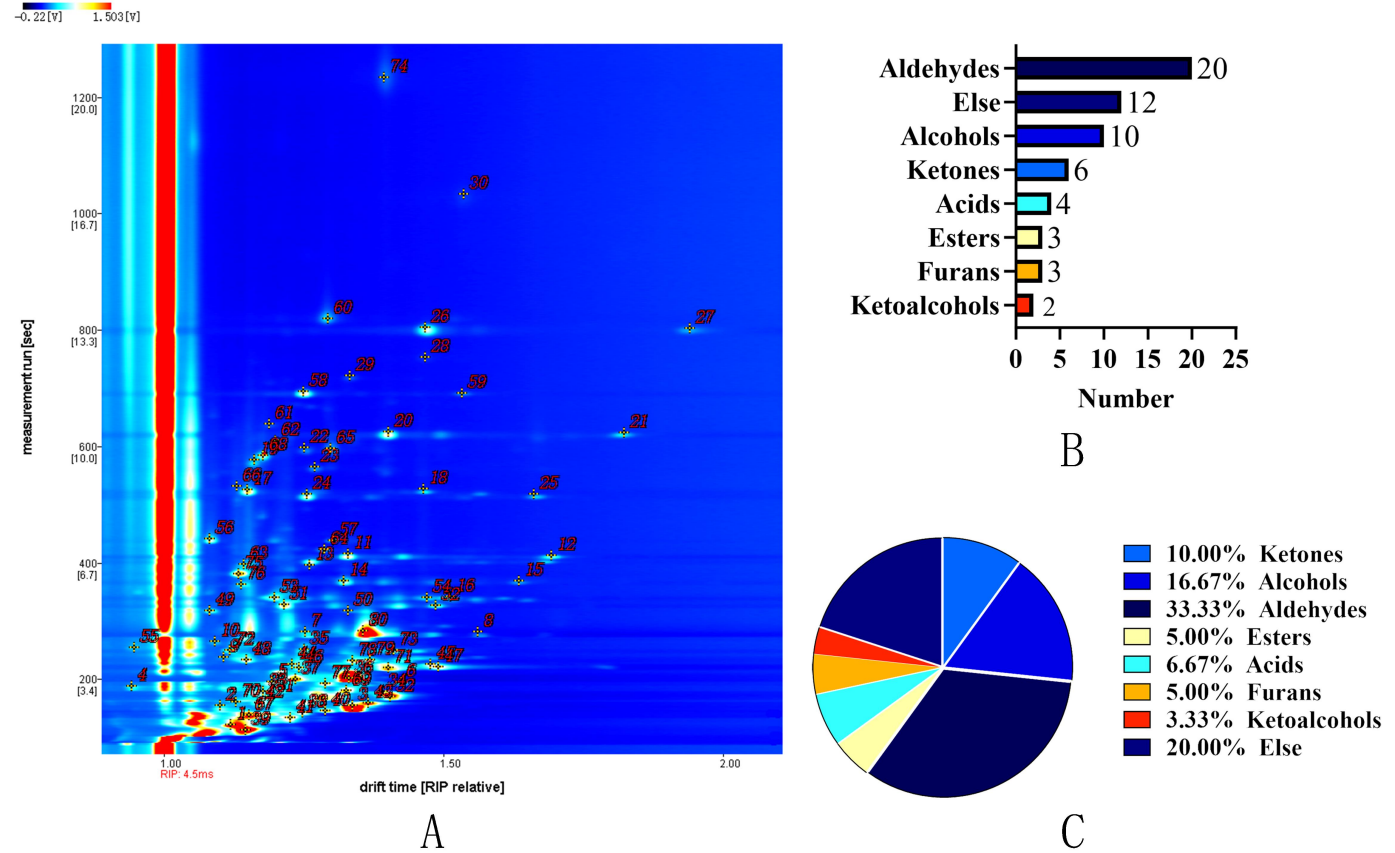
VOC profiles of prune preserves under different sugar impregnation methods: (**A**) total volatile compound content, (**B**) compound quantity, and (**C**) percentage distribution of volatile compound categories.

GC-IMS was employed to comprehensively characterize VOCs in prune preserves produced with three sugar impregnation methods. As shown in Figure 2 and Table 2, a total of 60 VOCs were detected, including 6 ketones (10%), 10 alcohols (16.67%), 20 aldehydes (33.33%), 3 esters (5%), 4 acids (6.67%), 3 furans (5%), 2 ketols (3.33%), and 12 unknown compounds. Among these, 48 VOCs were unambiguously identified in at least one processing method, with some compounds generating multiple ion peaks due to polymeric structures (monomers/dimers and trimers) [62]. Consistent with previous studies, aldehydes, alcohols, and ketones constituted the major volatile components of prune preserves [63].

**Figure 3:**
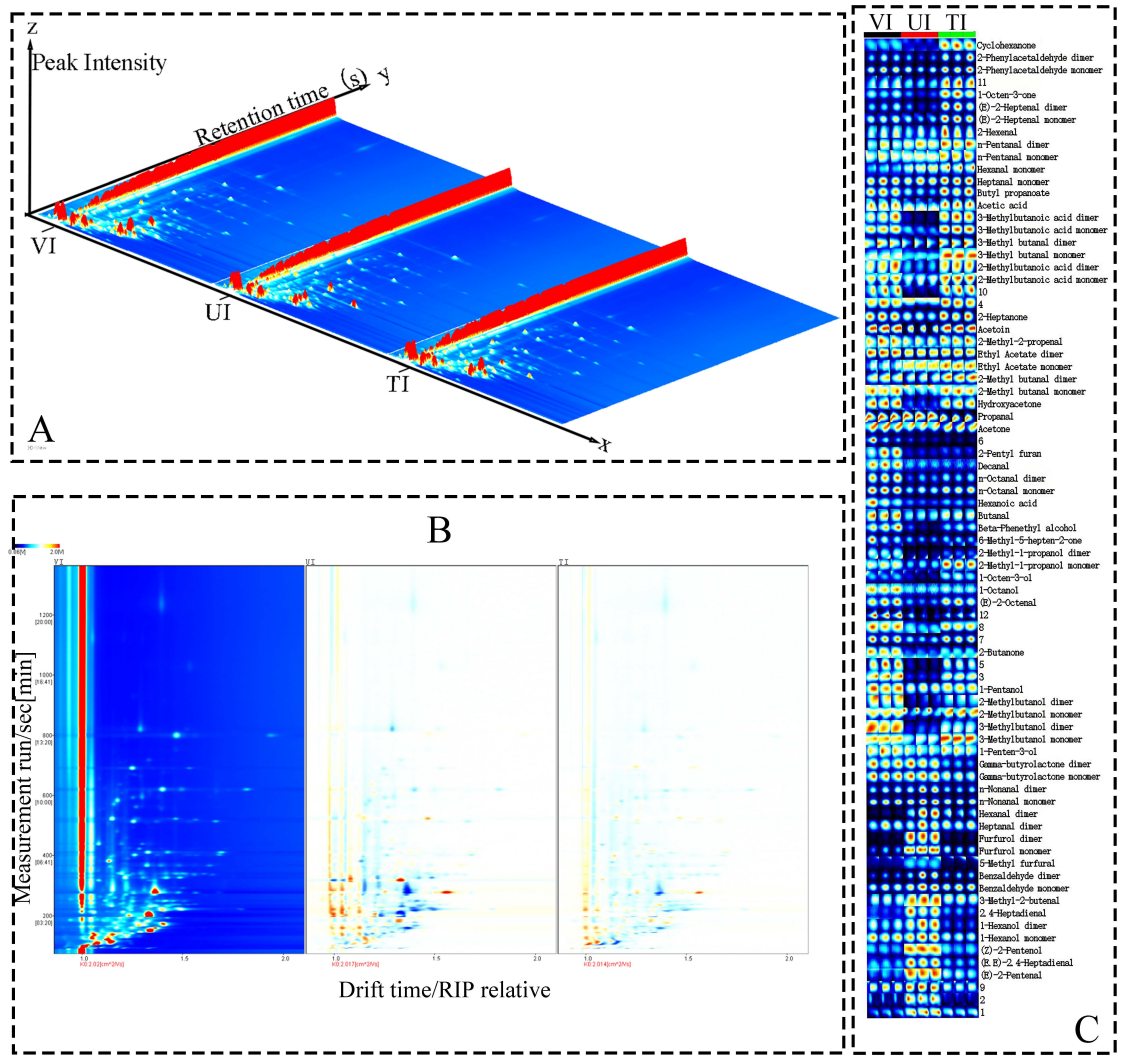
Comparison of volatile components in prune preserves via different sugar impregnation methods: 3D topographic map (**A**), topographic difference map (**B**), and fingerprint map (**C**).

As depicted in Figure 3A, with drift time as the X-axis, retention time as the Y-axis, and ion peak intensity as the Z-axis, red spots denote high-concentration VOCs, while white indicates low concentration. Figure 3B shows spectral subtraction with the VI group as the reference, where large blue areas in the UI and TI groups indicate lower volatile component concentrations than VI. Figure 3C reveals significant inter-group differences: aldehyde characteristic peaks in VI exhibit notably higher signal intensities than UI and TI, whereas UI shows more prominent alcohol peaks. The VI group shows significantly enhanced signal intensities of (E)-2-pentenal, (E,E)-2,4-heptadienal, (Z)-2-pentenol, hexanol, 2,4-heptadienal, 3-methyl-2-butenal, benzaldehyde, 5-methylfurfural, and furfural, conferring herbaceous, fruity, almond-like, and sweet notes typical of high-quality prune preserves [64]. This phenomenon is likely ascribed to two synergistic effects during VI processing: first, boiling point depression under vacuum conditions promotes thermal degradation of sugars and amino acids, and second, effective oxygen exclusion mitigates oxidative degradation of thermally generated flavor precursors [65]. In contrast, UI generated distinct volatile profiles dominated by alcohols and esters (e.g., butyrolactone, 1-penten-3-ol, 3-methylbutanol, 2-methylbutanol, pentanol, 2-butanone), imparting fresh fruity and floral notes but lacking flavor complexity due to low aldehyde content [66]. This may result from ultrasonic cavitation disrupting cell walls to release glycoside-bound aroma precursors and activating esterases to catalyze esterification [67]. The TI group showed the simplest volatile composition, with reduced overall aldehyde and alcohol intensities, attributed to >60% loss of heat-sensitive compounds and secondary degradation of primary aroma molecules under prolonged 90 °C exposure [68]. These results demonstrate that VI optimizes thermal degradation and oxidation reactions to efficiently retain and enrich characteristic aldehydes, making it the optimal process for shaping the authentic and complex aroma of prunes. This provides a theoretical basis for targeted flavor regulation in preserved fruit products.

#### 3.5.2. Multivariate Analysis of VOCs

**Figure 4:**
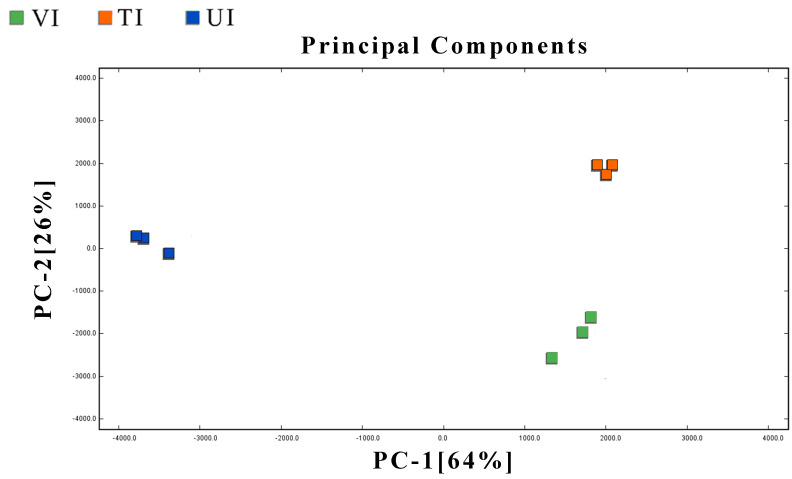
PCA of volatile components in prune preserves under different sugar impregnation methods.

**Figure 5:**
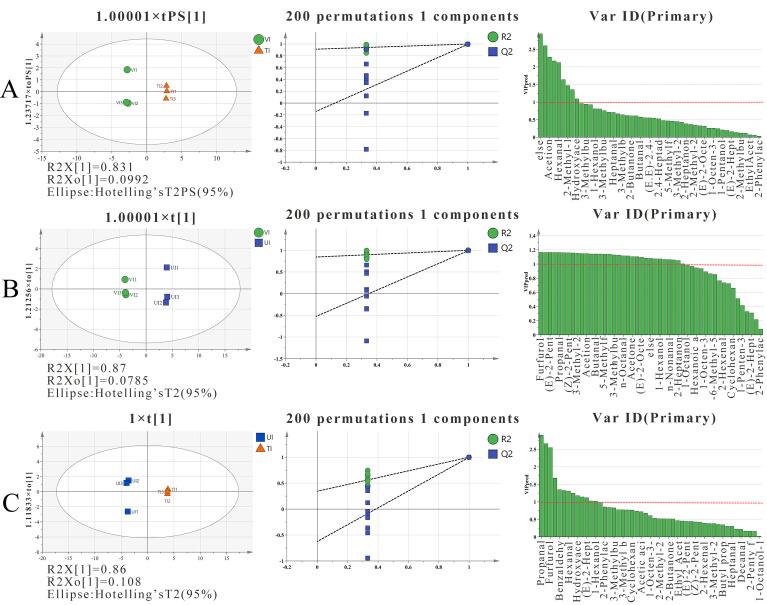
OPLS-DA score plot, displacement test verification, and VIP value score ranking of samples. (**A**) for VI vs. TI; (**B**) for VI vs.UI; (**C**) for UI vs. TI.

To effectively characterize and differentiate the flavor profiles of prune preserves produced by distinct sugar impregnation methods, multivariate statistical models were constructed using principal component analysis (PCA, Figure 4) and orthogonal partial least squares discriminant analysis (OPLS-DA, Figure 5) for sample classification and feature biomarker screening. The PCA model exhibited cumulative variances of 64% (PC1) and 26% (PC2), totaling 90%, with significant clustering separation among VI, TI, and UI groups, indicating that volatile differences induced by different processes were effectively distinguished via PCA. OPLS-DA models were further built to validate inter-group significance. Model parameters (e.g., Figure 5A for VI vs. TI, Figure 5B for VI vs. UI, Figure 5C for UI vs. TI) showed R^2^Y (goodness of fit) and Q^2^ (predictive ability) approaching 1, demonstrating excellent data interpretability and prediction accuracy. Following 200-cycle permutation tests, the permutation regression lines exhibited a “steep slope-intercept” feature (Q^2^ < 0), confirming no overfitting risk and remarkable model stability. Based on variable importance in projection (VIP > 1), key biomarkers were screened: Five compounds (e.g., Ethotoin, hexanal, 2-methyl-1-propanol) distinguished VI from TI, attributed to VI’s low-temperature vacuum environment inhibiting thermal degradation and enhancing retention of heat-sensitive VOCs like hexanal [69]. Seventeen compounds (e.g., furfural, (E)-2-pentenal, propionaldehyde) differentiated VI from UI, with significantly higher enrichment in VI due to vacuum-induced inhibition of thermal degradation, whereas the ultrasonic cavitation in UI accelerated furfural conversion to furanone derivatives [70]. Seven compounds (e.g., propionaldehyde, furfuryl alcohol, benzaldehyde) discriminated UI from TI, where TI promoted Maillard reactions, increasing benzaldehyde content relative to UI [71]. These multivariate analyses not only enabled precise classification of samples from different processes but also screened characteristic biomarkers from GC-IMS datasets, providing critical insights for flavor quality regulation in prune preserves processing.

### 3.6. E-Tongue Analysis

**Figure 6:**
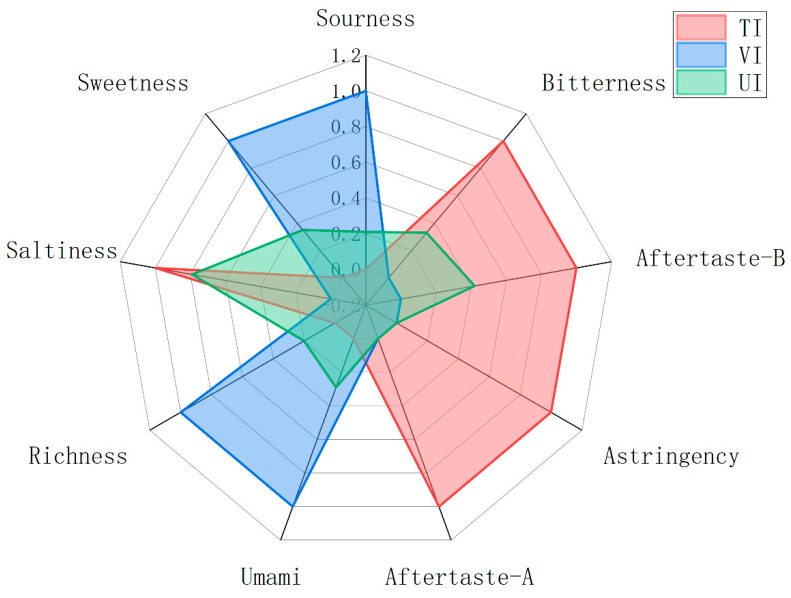
E-tongue analysis radar chart of prune preserves processed by different sugar impregnation methods.

To eliminate dimensional discrepancies among taste indices and facilitate visual comparison, raw data were normalized using Max-Min normalization. The normalized flavor radar chart (Figure 6) vividly illustrates the impact of different sugar impregnation methods on the overall taste profile of prune preserves. As shown in Figure 6, all treatment groups exhibited high normalized values for sourness, confirming it as the dominant basic flavor of prune preserves [72]. In contrast to UI and TI, the VI-treated samples showed significantly higher normalized values for sweetness and umami, indicating that VI more effectively promoted the penetration and enrichment of sugars and umami-related compounds (e.g., free amino acids, nucleotides) in the pulp [73]. Additionally, VI significantly reduced response values for bitterness, astringency, and aftertaste–bitterness, with some values approaching the instrument detection threshold, suggesting that this process inhibits the formation or migration of off-flavor compounds. Since the response of the sweetness sensor is related to the measured TSS value, the perceived taste differences may be more attributed to the change in sugar concentration rather than the mere phenolic bitterness [74]. The higher TSS value in the VI group directly led to an increase in its sweetness intensity, while the lower sugar content in the UI and TI samples might have amplified the perception of bitterness and astringency caused by residual phenols [75]. The core advantage of VI resides in its vacuum negative-pressure environment, which first expels air from pulp tissues to reduce sugar solution penetration resistance. Upon atmospheric pressure recovery, the pressure difference drives faster and more uniform sugar solution infiltration, accelerating mass transfer. This not only increases sweet substance content but also promotes the enrichment of natural umami components [36]. Unlike TI’s high-temperature process, VI operates at lower temperatures, retaining sourness, reducing bitterness and astringency, and inhibiting flavor degradation. Sourness retention: low temperatures reduce the volatilization and thermal decomposition of heat-sensitive organic acids, preserving the natural sourness of prune preserves [76]. Reduced bitterness and astringency formation: low-temperature and low-oxygen partial pressure inhibit polyphenol oxidase (PPO) activity, minimizing phenolic oxidation polymerization and controlling the generation of bitter and astringent substances at the source [77]. Flavor degradation inhibition: the mild thermal environment significantly retards enzymatic browning and other heat-induced oxidative degradation, effectively preserving the color, natural flavor, and volatile components of prune preserves [78].

## 4. Conclusions

This study systematically evaluated sugar impregnation processes for prune preserves, focusing on the impacts of VI, UI, and TI on comprehensive product quality. Results demonstrate that VI exhibited optimal performance in maintaining flavor, preserving nutrients, and optimizing texture. GC-IMS analysis detected 60 VOCs. The VI-treated prune preserves exhibited the most abundant volatile profiles, with key characteristic flavor compounds including (E)-2-pentenal, (E,E)-2,4-heptadienal, (Z)-2-pentenol, hexanol, 2,4-heptadienal, and furfural. These compounds collectively contributed to the product’s unique herbaceous, fruity, almond-like, and sweet notes, significantly enhancing flavor complexity and sensory appeal. VI treatment significantly enhanced the retention of key quality indicators. The TA content (0.77 ± 0.05%) was notably higher than that in the UI (0.63 ± 0.01%; 22.2% increase) and TI (0.53 ± 0.03%; 33.96% increase) groups, exhibiting a positive correlation with the sourness intensity detected by the E-tongue. TPC and TFC contents reached 1.23 ± 0.07 mg GAE/g and 17.55 ± 0.81 mg RE/g, respectively. In terms of color, the VI group exhibited a significantly lower BI (0.37 ± 0.03) than the TI group (0.83 ± 0.03). Furthermore, the lightness values of the VI group were increased by 13.55% and 26.54% compared with the UI and TI groups, respectively, effectively maintaining the natural color appearance of prune preserves, which had a positive impact on market acceptability. Additionally, the hardness (182.56 ± 5.23 N) and chewiness (126.34 ± 4.17 mJ) of the VI-treated prune preserves were significantly enhanced compared with the UI and TI groups. This optimized texture not only endowed the product with an ideal balance of hardness and chewiness but also better aligned with consumer preferences for preserves with appropriate bite elasticity. In conclusion, under the conditions of this study, the VI group demonstrated the highest TSS and ideal texture parameters. Its flavor and color retention effects were superior to those of the UI and TI groups, indicating that VI has good application potential in the industrial production of preserved fruits.

## Figures and Tables

**Table 1:** The TSS, a_w_, color (L*, a*, b*), and TPA of prune preserves by three sugar impregnation methods.

Indicators	OD Techniques		
	VI	UI	TI
TSS (°Brix)	30.10 ± 0.26 ^a^	27.47 ± 0.60 ^b^	23.47 ± 0.71 ^c^
a_w_	0.86 ± 0.01 ^c^	0.91 ± 0.01 ^b^	0.93 ± 0.01 ^a^
L*	31.85 ± 1.56 ^a^	28.05 ± 0.83 ^b^	25.17 ± 0.85 ^c^
a*	17.03 ± 0.58 ^a^	12.88 ± 0.72 ^b^	11.13 ± 0.95 ^c^
b*	16.88 ± 0.16 ^b^	17.68 ± 0.63 ^b^	18.79 ± 0.36 ^a^
Hardness (g)	187.63 ± 4.04 ^b^	176.53 ± 5.81 ^b^	156.25 ± 4.55 ^a^
Springiness (mm)	0.82 ± 0.03 ^b^	0.73 ± 0.02 ^ab^	0.62 ± 0.06 ^a^
Chewiness (mJ)	113.99 ± 6.61 ^c^	93.52 ± 5.70 ^b^	69.56 ± 4.65 ^a^
Cohesiveness	0.80 ± 0.04 ^b^	0.72 ± 0.02 ^ab^	0.67 ± 0.03 ^a^
Gumminess	129.38 ± 6.42 ^a^	106.53 ± 4.27 ^ab^	84.05 ± 6.25 ^a^

Note: Different letters within the same column indicate significant differences (*p* < 0.05). The initial TSS of the raw fruit is 18.5 ± 0.5 °Brix.

**Table 2:** The different volatile components of prune preserves made with three sugar impregnation methods.

No.	Compound	CAS#	Formula	MW	(RI)	Rt [s]	Dt [a.u.]
1	Acetone	C67641	C_3_H_6_O	58.1	529.6	122.764	1.12109
2	Ethyl Acetate monomer	C141786	C_4_H_8_O_2_	88.1	613.2	154.71	1.10222
3	Ethyl Acetate dimer	C141786	C_4_H_8_O_2_	88.1	613.2	154.71	1.33808
4	1-Penten-3-ol	C616251	C_5_H_10_O	86.1	683.5	187.733	0.94289
5	n-Pentanal monomer	C110623	C_5_H_10_O	86.1	695.6	195.271	1.19342
6	n-Pentanal dimer	C110623	C_5_H_10_O	86.1	696.1	195.63	1.42404
7	Hexanal monomer	C66251	C_6_H_12_O	100.2	797.2	282.135	1.25317
8	Hexanal dimer	C66251	C_6_H_12_O	100.2	796.1	281.059	1.56346
9	(E)-2-Pentenal	C1576870	C_5_H_8_O	84.1	750.4	238.344	1.10746
10	3-Methyl-2-butenal	C107868	C_5_H_8_O	84.1	780.6	265.983	1.09174
11	Heptanal monomer	C111717	C_7_H_14_O	114.2	904.8	415.906	1.33096
12	Heptanal dimer	C111717	C_7_H_14_O	114.2	903.2	413.173	1.69531
13	2-Heptanone	C110430	C_7_H_14_O	114.2	893.1	396.777	1.26165
14	1-Hexanol monomer	C111273	C_6_H_14_O	102.2	872.6	368.767	1.32208
15	1-Hexanol dimer	C111273	C_6_H_14_O	102.2	872.6	368.767	1.63666
16	2-Hexenal	C505577	C_6_H_10_O	98.1	852	342.806	1.51403
17	Benzaldehyde monomer	C100527	C_7_H_6_O	106.1	963.9	526.58	1.14967
18	Benzaldehyde dimer	C100527	C_7_H_6_O	106.1	964.2	527.263	1.46604
19	1-Octen-3-ol	C3391864	C_8_H_16_O	128.2	986.5	576.452	1.16212
20	n-Octanal monomer	C124130	C_8_H_16_O	128.2	1011.9	624.957	1.40205
21	n-Octanal dimer	C124130	C_8_H_16_O	128.2	1011.1	623.591	1.82506
22	2-Pentyl furan	C3777693	C_9_H_14_O	138.2	995.8	598.314	1.25276
23	1-Octen-3-one	C4312996	C_8_H_14_O	126.2	981.7	565.521	1.27053
24	(E)-2-Heptenal monomer	C18829555	C_7_H_12_O	112.2	960	518.382	1.25631
25	(E)-2-Heptenal dimer	C18829555	C_7_H_12_O	112.2	960	518.382	1.66332
26	n-Nonanal monomer	C124196	C_9_H_18_O	142.2	1106.9	804.46	1.46895
27	n-Nonanal dimer	C124196	C_9_H_18_O	142.2	1106.5	803.747	1.9419
28	1-Octanol	C111875	C_8_H_18_O	130.2	1082	753.088	1.46895
29	(E)-2-Octenal	C2548870	C_8_H_14_O	126.2	1065.7	720.98	1.3341
30	Decanal	C112312	C_10_H_20_O	156.3	1201	1033.035	1.53767
31	3-Methyl butanal monomer	C590863	C_5_H_10_O	86.1	649.5	170.974	1.19066
32	3-Methyl butanal dimer	C590863	C_5_H_10_O	86.1	649.5	170.974	1.40516
33	2-Methyl butanal monomer	C96173	C_5_H_10_O	86.1	667.8	179.786	1.17906
34	2-Methyl butanal dimer	C96173	C_5_H_10_O	86.1	666.9	179.366	1.39212
35	1-Pentanol	C71410	C_5_H_12_O	88.1	764.7	251.007	1.25226
36	Acetoin	C513860	C_4_H_8_O_2_	88.1	704.3	201.526	1.33233
37	Hydroxyacetone	C116096	C_3_H_6_O_2_	74.1	702.6	200.302	1.23621
38	2-Butanone	C78933	C_4_H_8_O	72.1	583.7	142.622	1.24876
39	Propanal	C123386	C_3_H_6_O	58.1	499.9	113.056	1.14671
40	Butanal	C123728	C_4_H_8_O	72.1	593.5	146.532	1.28983
41	2-Methyl-2-propenal	C78853	C_4_H_6_O	70.1	563.4	134.803	1.22636
42	2-Methyl-1-propanol monomer	C78831	C_4_H_10_O	74.1	625.4	159.971	1.17285
43	2-Methyl-1-propanol dimer	C78831	C_4_H_10_O	74.1	625.4	159.971	1.36699
44	2-Methylbutanol monomer	C137326	C_5_H_12_O	88.1	736.4	226.467	1.23023
45	2-Methylbutanol dimer	C137326	C_5_H_12_O	88.1	736.4	226.467	1.47787
46	3-Methylbutanol monomer	C123513	C_5_H_12_O	88.1	729.3	220.739	1.24255
47	3-Methylbutanol dimer	C123513	C_5_H_12_O	88.1	730.9	221.967	1.49155
48	Furfurol monomer	C98011	C_5_H_4_O_2_	96.1	831.3	318.529	1.08247
49	Furfurol dimer	C98011	C_5_H_4_O_2_	96.1	830.6	317.711	1.33011
50	3-Methylbutanoic acid monomer	C503742	C_5_H_10_O_2_	102.1	839.9	328.349	1.21655
51	3-Methylbutanoic acid dimer	C503742	C_5_H_10_O_2_	102.1	838.5	326.712	1.48745
52	2-Methylbutanoic acid monomer	C116530	C_5_H_10_O_2_	102.1	849.9	340.215	1.19877
53	2-Methylbutanoic acid dimer	C116530	C_5_H_10_O_2_	102.1	849.9	340.215	1.4724
54	(Z)-2-Pentenol	C1576950	C_5_H_10_O	86.1	768.7	254.7	0.94702
55	γ-butyrolactone monomer	C96480	C_4_H_6_O_2_	86.1	919.5	440.908	1.08266
56	γ-butyrolactone dimer	C96480	C_4_H_6_O_2_	86.1	918.1	438.46	1.30323
57	2-Phenylacetaldehyde monomer	C122781	C_8_H_8_O	120.2	1051.3	693.99	1.25011
58	2-Phenylacetaldehyde dimer	C122781	C_8_H_8_O	120.2	1050.3	692.223	1.53526
59	β-Phenethyl alcohol	C60128	C_8_H_10_O	122.2	1114.2	820.361	1.29427
60	(E,E)-2,4-Heptadienal	C4313035	C_7_H_10_O	110.2	1019.8	638.316	1.18941
61	2,4-Heptadienal	C5910850	C_7_H_10_O	110.2	1002.8	610.038	1.20044
62	Cyclohexanone	C108941	C_6_H_10_O	98.1	894.1	398.438	1.14433
63	Butyl propanoate	C590012	C_7_H_14_O_2_	130.2	909.3	423.463	1.28891
64	Hexanoic acid	C142621	C_6_H_12_O_2_	116.2	995.1	596.667	1.29888
65	5-Methyl furfural	C620020	C_6_H_6_O_2_	110.1	966.2	531.469	1.13103
66	Acetic acid	C64197	C_2_H_4_O_2_	60.1	573.6	138.681	1.15396
67	6-Methyl-5-hepten-2-one	C110930	C_8_H_14_O	126.2	989.9	584.397	1.17773
68	Unidentified compound 1	*	*	*	669.7	180.716	1.3274
69	Unidentified compound 2	*	*	*	628	161.13	1.12899
70	Unidentified compound 3	*	*	*	728.5	220.082	1.40308
71	Unidentified compound 4	*	*	*	764.3	250.608	1.11941
72	Unidentified compound 5	*	*	*	763.4	249.79	1.4122
73	Unidentified compound 6	*	*	*	1268	1234.62	1.3949
74	Unidentified compound 7	*	*	*	882	381.315	1.13435
75	Unidentified compound 8	*	*	*	868.7	363.734	1.13896
76	Unidentified compound 9	*	*	*	693	193.415	1.2893
77	Unidentified compound 10	*	*	*	742.8	231.812	1.33913
78	Unidentified compound 11	*	*	*	745.2	233.886	1.37029
79	Unidentified compound 12	*	*	*	798	282.984	1.35759

Note: The asterisk (*) indicates an unknown.

## Data Availability

The original contributions presented in this study are included in the article. Further inquiries can be directed to the corresponding author.

## References

[1] Savic I.M., Savic-Gajic I.M. (2020). Optimization study on extraction of antioxidantsfrom plum seeds (*Prunus domestica* L.). Optim. Eng..

[2] Ma Y.Y., Zhang W.D., Cheng S.B., Liu Y.X., Yang W.T., Wang Y., Guo M.R., Chen G.G. (2022). Postharvest storage at near-freezing temperature maintained the quality and antioxidant properties of *Prunus domestica* L. cv. Ximei fruit. Sci. Hortic..

[3] Li L., Yu Y.Y., Xu Y.J., Wu J.J., Yu W.X., Peng J., An K.J., Zou B., Yang W.Y. (2021). Effect of ultrasound-assisted osmotic dehydration pretreatment on the drying characteristics and quality properties of Sanhua plum (*Prunus salicina* L.). LWT.

[4] Almeida A.R., Mussi L.P., Oliveira D.B., Pereira N.R. (2014). Effect of Temperature and Sucrose Concentration on the Retention of Polyphenol Compounds and Antioxidant Activity of Osmotically Dehydrated Bananas. J. Food Process. Preserv..

[5] Rahaman A., Zeng X.A., Kumari A., Rafiq M., Siddeeg A., Manzoor M.F., Baloch Z., Ahmed Z. (2019). Influence of ultrasound-assisted osmotic dehydration on texture, bioactive compounds and metabolites analysis of plum. Ultrason. Sonochem..

[6] Zhang W. (2016). Simple Technology and Low Threshold: The Preserved Fruit Industry Needs to Develop into “Healthy Food”. China Food.

[7] Zhu M., Liu D.B., Yang X.Y., Jing W.N. (2025). Sugar Infiltration Methods on the Quality Characteristics of Preserved Passion Fruit. J. Shandong Agric. Univ. (Nat. Sci. Ed.).

[8] Cui S.C., Xiang F.X., Lu H.X. (2018). The Influence of Sugar Preservation Technology on the Sugar Crystallization Phenomenon of Ganyingzi and Its Optimization. Zhejiang Agric. Sci..

[9] Neri L., Di-Biase L., Sacchetti G., Mattia C.D., Santarelli V., Mastrocola D., Pittia P. (2016). Use of vacuum impregnation for the production of high quality fresh-like apple products. J. Food Eng..

[10] Ji X.L., Guo J.H., Tian J.Y., Ma K., Liu Y.Q. (2023). Research progress on degradation methods and product properties of plant polysaccharides. J. Light Ind..

[11] Zhang Y.W., Abatzoglou N. (2020). Review: Fundamentals, applications and potentials of ultrasound-assisted drying. Chem. Eng. Res. Des..

[12] Ji X.L., Hou C.Y., Yan Y.Z., Shi M.M., Liu Y.Q. (2020). Comparison of structural characterization and antioxidant activity of polysaccharides from jujube (*Ziziphus jujuba Mill*.) fruit. Int. J. Biol. Macromol..

[13] Yan S.K., Dong R., Yang J.P., Wang G.Q. (2024). Objective Quantification Technique and Widely Targeted Metabolomics-Based Analysis of the Effects of Different Saccharidation Processes on Preserved French Plums. Molecules.

[14] Fabela-Morón M.F. (2024). Bioactive compounds, sensory attributes, and flavor perceptions involved in taste-active molecules in fruits and vegetables. Front. Nutr..

[15] Yuan J.J., Li H.B., Cao S.Q., Liu Z.B., Li N., Xu D., Mo H.Z., Hu L.B. (2023). Monitoring of Volatile Compounds of Ready-to-Eat Kiwifruit Using GC-IMS. Foods.

[16] Xuan X.T., Sun R.Y., Zhang X.Y., Cui Y., Lin X.D., Sun Y., Deng W., Liao X.J., Ling J.J. (2022). Novel application of HS-GC-IMS with PCA for characteristic fingerprints and flavor compound variations in NFC Chinese bayberry (*Myrica rubra*) juice during storage. LWT.

[17] Gu S., Zhang J., Wang J., Wang X.Y., Du D.D. (2021). Recent development of HS-GC-IMS technology in rapid and non-destructive detection of quality and contamination in agri-food products. Trends Anal. Chem..

[18] Aouadi B., Zaukuu J.L.Z., Vitális F., Bodor Z., Fehér O., Gillay Z., Bazar G., Kovacs Z. (2020). Historical evolution and food control achievements of near infrared spectroscopy, electronic nose, and electronic tongue—Critical overview. Sensors.

[19] De-Roos K.B. (2003). Effect of texture and microstructure on flavour retention and release. Int. Dairy J..

[20] Yu Z.T., Deng J., Ma N., Sun Y., Wang J., Liu J.M., Zhang Y., Lu Y.S., Wang S. (2025). Comparative analysis of quality, structural, and flavor alterations in chestnuts (Castanea mollissima Blume) subjected to different thermal processing techniques. Food Chem..

[21] Gautam S., Kathuria D., Hamid, Dobhal A., Singh N. (2024). Vacuum impregnation: Effect on food quality, application and use of novel techniques for improving its efficiency. Food Chem..

[22] Zhou X., Li R., Lyng J.G., Wang S.J. (2018). Dielectric Properties of Kiwifruit Associated with a Combined Radio Frequency Vacuum and Osmotic Drying. J. Food Eng..

[23] Pantelidou D., Gerogiannis K., Goula A., Gonas C. (2021). Ultrasound-Assisted Osmotic Dehydration as a Method for Supplementing Potato with Unused Chokeberries Phenolics. Food Bioprocess Technol..

[24] Kaur D., Singh M., Zalpouri R., Singh I. (2022). Osmotic dehydration of fruits using unconventional natural sweeteners and non-thermal-assisted technologies: A review. J. Food Process. Preserv..

[25] Yu Z.T., Lu Y.S., Wei F., Zhang Y., Wang S., Dong L. (2024). The impact of natural spices additions on hazards development and quality control in roast beef patties. Food Chem..

[26] Liu D.Y., Guo W.C. (2017). Nondestructive determination of soluble solids content of persimmons by using dielectric spectroscopy. Int. J. Food Prop..

[27] Xie J.W., Ni Z.P., Guo M.Y., Song X.L. (2023). Sterilization Effect and Quality Change Analysis of Preserved Fruits Treated by Microwave and Ozone. Mod. Food Sci. Technol..

[28] Paul V., Singh A., Pandey R. (2010). Determination of titrable acidity (TA). Postharvest Biol. Technol..

[29] Lee B., Seo J.D., Rhee J.K., Kim C.Y. (2016). Heated Apple Juice Supplemented with Onion Has Greatly Improved Nutritional Quality and Browning Index. Food Chem..

[30] López A., Pittori A., Di-Sarli A.Y. (2024). Architectural self-compacting concretes and their color stability for 10 years. Constr. Build. Mater..

[31] Dróżdż P., Šėžienė V., Wójcik J., Pyrzyńska K., Pyrzynska K. (2018). Evaluation of Bioactive Compounds, Minerals and Antioxidant Activity of Lingonberry (*Vaccinium vitis-idaea* L.) Fruits. Molecules.

[32] Ghasemzadeh A., Jaafar H.Z.E., Rahmat A. (2016). Variation of the Phytochemical Constituents and Antioxidant Activities of Zingiber officinale var. rubrum Theilade Associated with Different Drying Methods and Polyphenol Oxidase Activity. Molecules.

[33] Zahari I., Ferawati F., Helstad A., Ahlstrm C., Östbring K., Rayner M., Purhagen J.K. (2020). Development of High-Moisture Meat Analogues with Hemp and Soy Protein Using Extrusion Cooking. Foods.

[34] Zhang D., Ji H.W., Liu S.C., Gao J. (2020). Similarity of aroma attributes in hot-air-dried shrimp (*Penaeus vannamei*) and its different parts using sensory analysis and GC-MS. Food Res. Int..

[35] Bai J., Fan Y., Zhu L., Wang Y., Hou H. (2022). Characteristic Flavor of Antarctic Krill (*Euphausia Superba*) and White Shrimp (*Penaeus Vannamei*) Induced by Thermal Treatment. Food Chem..

[36] Zhao Y.Y., Xie J. (2004). Practical applications of vacuum impregnation in fruit and vegetable processing. Trends Food Sci. Tech..

[37] Bich L., Pradeu T., Moreau J.F. (2019). Understanding Multicellularity: The Functional Organization of the Intercellular Space. Front. Physiol..

[38] Betoret E., Betoret N., Rocculi P., Dalla Rosa M. (2015). Strategies to improve food functionality: Structure-property relationships on high pressures homogenization, vacuum impregnation and drying technologies. Trends Food Sci. Technol..

[39] Ebrahimi N., Sadeghi R. (2017). Soluting-in and soluting-out of water-soluble polymers inaqueous carbohydrate solutions studied by vaporpressure osmometry. J. Mol. Liq..

[40] Barać G., Mastilović J., Kevrešan Ž., Milić B., Kovač R., Milović M., Keserović Z. (2022). Effects of Plant Growth Regulators on Plum (*Prunus Domestica* L.) Grown on Two Rootstocks at Harvest and at the Postharvest Period. Horticulturae.

[41] Wolf J., Göttingerová M., Kaplan J., Kiss T., Venuta R., Nečas T. (2020). Determination of the pomological and nutritional properties of selected plum cultivars and minor fruit species. Hort. Sci..

[42] Huang X., Wang H.K., Luo W.J., Xue S., Hayat F., Gao Z.H. (2021). Prediction of loquat soluble solids and titratable acid content using fruit mineral elements by artificial neural network and multiple linear regression. Sci. Hortic..

[43] Panayampadan A.S., Alam M.S., Aslam R., Kaur J. (2022). Vacuum impregnation process and its potential in modifying sensory, physicochemical and nutritive characteristics of food products. Food Eng. Rev..

[44] Sadras V.O., Petrie P.R., Moran M.A. (2012). Effects of elevated temperature in grapevine. II juice pH, titratable acidity and wine sensory attributes. Aust. J. Grape Wine Res..

[45] Obenland D., Collin S., Mackey B., Sievert J., Arpaia M.L. (2011). Storage temperature and time influences sensory quality of mandarins by altering soluble solids, acidity and aroma volatile composition. Postharvest Biol. Technol..

[46] Dias L.G., Sequeira C., Veloso A.C., Sousa M.E., Peres A.M. (2014). Evaluation of healthy and sensory indexes of sweetened beverages using an electronic tongue. Anal. Chim. Acta..

[47] Tsikrika K., Tzima K., Rai D.K. (2022). Recent advances in anti-browning methods in minimallyprocessed potatoes—A review. J. Food Process. Preserv..

[48] Nath P., Pandey N., Samota M., Sharma K., Kale S., Kannaujia P., Chauhan O.P., Saxena D.C. (2022). Browning Reactions in Foods. Advances in Food Chemistry: Food Components, Processing and Preservation.

[49] Qu W.J., Ruan W.Y., Feng Y.H., Liu Y., Tuly J.A., Zhou C.S. (2025). Assessing the dry inactivation mechanism of polyphenol oxidase (PPO) and peroxidase (POD) employing catalytic infrared treatment based on experiments and molecular simulations. Innov. Food Sci. Emerg. Technol..

[50] Min T., Xie J., Zheng M.L., Yi Y., Hou W.F., Wang L.M., Ai Y.W., Wang H.X. (2017). The effect of different temperatures on browning incidence and phenol compound metabolism in fresh-cut lotus (Nelumbo nucifera G.) root. Postharvest Biol. Technol..

[51] Gómez-Martínez H., Bermejo A., Zuriaga E., Badenes M.L. (2021). Polyphenol content in apricot fruits. Sci. Hortic..

[52] Réblová Z. (2012). Effect of temperature on the antioxidant activity of phenolic acids. Czech J. Food Sci..

[53] Mustafa A.M., Mazzara E., Abouelenein D., Angeloni S., Nunez S., Sagratini G., Maggi F. (2022). Optimization of solvent-free microwave-assisted hydrodiffusion and gravity extraction of Morus nigra L. fruits maximizing polyphenols, sugar content, and biological activities using central composite design. Pharmaceuticals.

[54] Aaby K., Amundsen M.R. (2023). The stability of phenolic compounds and the colour of lingonberry juice with the addition of different sweeteners during thermal treatment and storage. Heliyon.

[55] Zheng Y.Z., Deng G., Zhang Y.C. (2022). Multiple free radical scavenging reactions of flavonoids. Dyes Pigm..

[56] Daskalaki D., Kefi G., Kotsiou K., Tasioula-Margari M. (2009). Evaluation of phenolic compounds degradation in virgin olive oil during storage and heating. J. Food Nutr. Res..

[57] Schaefer H.M. (2011). Why fruits go to the dark side. Acta Oecol..

[58] Liu Q.Y., Lin Z.Q., Chen X.M., Chen J.W., Wu J.S., Chen H.G., Zeng X.F. (2022). Characterization of structures and gel properties of ultra-high-pressure treated-myofibrillar protein extracted from mud carp (*Cirrhinus molitorella*) and quality characteristics of heat-induced sausage products. LWT.

[59] Li C.S., Cui Q.Y., Li L.H., Huang H., Chen S.J., Zhao Y.Q., Wang Y. (2024). Formation and improvement mechanism of physical property and volatile flavor of fermented tilapia surimi by newly isolated lactic acid bacteria based on two dimensional correlation networks. Food Chem..

[60] Ruan J.W., Xue G., Liu Y., Ye B., Li M., Xu Q. (2025). Optimization of the Vacuum Microwave Drying of Tilapia Fillets Using Response Surface Analysis. Foods..

[61] Nath K.G., Pandiselvam R., Sunil C.K. (2023). High-pressure processing: Effect on textural properties of food-A review. J. Food Eng..

[62] Li M.Q., Yang R.W., Zhang H., Wang S.L., Chen D., Lin S.Y. (2019). Development of a flavor fingerprint by HS-GC-IMS with PCA for volatile compounds of Tricholoma matsutake Singer. Food Chem..

[63] Abbas F., Zhou Y., O’Neill-Rothenberg D., Alam I., Ke Y., Wang H.C. (2023). Aroma components in horticultural crops: Chemical diversity and usage of metabolic engineering for industrial applications. Plants.

[64] Yu X.Y., Chen X.C., Li Y., Li L. (2022). Effect of Drying Methods on Volatile Compounds of Citrus Reticulata Ponkan and Chachi Peels as Characterized by GC-MS and GC-IMS. Foods.

[65] Johnson D.R., Decker E.A. (2015). The role of oxygen in lipid oxidation reactions: A review. Annu. Rev. Food Sci. Technol..

[66] Man L.M., Ren W., Sun M.G., Du Y.R., Chen H., Qin H.X., Chai W.Q., Zhu M.X., Liu G.Q., Wang C.F. (2023). Characterization of donkey-meat flavor profiles by GC-IMS and multivariate analysis. Front. Nutr..

[67] Song X., Capanoglu E., Simal-Gandara J., Chen F., Xiao J., Simal-Gandara J. (2022). Different Food Processing Technologies: A General Background. Retention of Bioactives in Food Processing.

[68] Oliveira S.M., Brandao T.R., Silva C.L. (2015). Influence of drying processes and pretreatments on nutritional and bioactive characteristics of dried vegetables: A review. Food Eng. Rev..

[69] Mahanta B.P., Bora P.K., Kemprai P., Borah G., Lal M., Haldar S. (2021). Thermolabile Essential Oils, Aromas and Flavours: Degradation Pathways, Effect of Thermal Processing and Alteration of Sensory Quality. Food Res. Int..

[70] Saleena P., Jayashree E., Anees K. (2023). A Comprehensive Review on Vacuum Impregnation: Mechanism, Applications and Prospects. Food Bioprocess Technol..

[71] Mao C., Chen Y.R., Ye P.F., Chang Z., Sun S.J., Liu R., Wang Y.Q., Chen X.W., Fu H.F., Wang Y.Y. (2025). Sugar boiling pre-treatment improves radio frequency explosion puffing quality on modifying the physicochemical and functional properties of purple sweet potato flour. Int. J. Biol. Macromol..

[72] Stacewicz-Sapuntzakis M., Bowen P.E., Hussain E.A., Damayanti-Wood B.I., Farnsworth N.R. (2001). Chemical composition and potential health effects of prunes: A functional food?. Crit. Rev. Food Sci. Nutr..

[73] Xia R.J., Qiao Y.T., Xu H., Hou H.S., Qian G.L., Wang Y.F., Li Y.T., Yan M., Pan S., Xin G. (2024). Unlocking the Potential of the Umami Taste-Presenting Compounds: A Review of the Health Benefits, Metabolic Mechanisms and Intelligent Detection Strategies. Food Rev. Int..

[74] Pagliarini E., Proserpio C., Spinelli S., Lavelli V., Laureati M., Arena E., Dinnella C. (2021). The role of sour and bitter perception in liking, familiarity and choice for phenol-rich plant-based foods. Food Qual. Prefer..

[75] Huang R., Xu C. (2022). An overview of the perception and mitigation of astringency associated with phenolic compounds. Compr. Rev. Food Sci. Food Saf..

[76] ElGamal R., Song C., Rayan A.M., Liu C., Al-Rejaie S., ElMasry G. (2023). Thermal degradation of bioactive compounds during drying process of horticultural and agronomic products: A comprehensive overview. Agronomy.

[77] Buckow R., Weiss U., Knorr D. (2009). Inactivation kinetics of apple polyphenol oxidase in different pressure–temperature domains. Innov. Food Sci. Emerg. Technol..

[78] Mu Y.W., Zhang B.H., Zeng C.Z., Zhu T.D., Hu S.H. (2025). Mechanistic and Multi-Parametric Insights into Preserving Nutritional, Bioactive, and Flavor Attributes of Daylily (*Hemerocallis citrina*): A Comparative Evaluation of Freeze-Drying, Hot-Air Drying, and Sun Drying. LWT.

